# Exploring and modelling colon cancer inter-tumour heterogeneity: opportunities and challenges

**DOI:** 10.1038/s41389-020-00250-6

**Published:** 2020-07-09

**Authors:** Joyce Y. Buikhuisen, Arezo Torang, Jan Paul Medema

**Affiliations:** 1 Laboratory for Experimental Oncology and Radiobiology, Center for Experimental Molecular Medicine, Cancer Center Amsterdam, Amsterdam UMC, University of Amsterdam, Amsterdam, The Netherlands; 2 Oncode Institute, Amsterdam, The Netherlands

**Keywords:** Colorectal cancer, Cancer models, Targeted therapies, Cancer therapeutic resistance, Chemotherapy

## Abstract

Colon cancer inter-tumour heterogeneity is installed on multiple levels, ranging from (epi)genetic driver events to signalling pathway rewiring reflected by differential gene expression patterns. Although the existence of heterogeneity in colon cancer has been recognised for a longer period of time, it is sparingly incorporated as a determining factor in current clinical practice. Here we describe how unsupervised gene expression-based classification efforts, amongst which the consensus molecular subtypes (CMS), can stratify patients in biological subgroups associated with distinct disease outcome and responses to therapy. We will discuss what is needed to extend these subtyping efforts to the clinic and we will argue that preclinical models recapitulate CMS subtypes and can be of vital use to increase our understanding of treatment response and resistance and to discover novel targets for therapy.

## Introduction

When diagnosed at early stages, colon cancer is associated with good overall survival rates, but these numbers rapidly decline in stage III or stage IV metastatic disease^1^. The high heterogeneity of colon cancer on the genetic and gene regulatory level contributes to differences in therapy response and, consequently, survival^2^. In an era where (semi-)individualised treatment can be more readily achieved due to advancements and cost reductions in various molecular biology techniques, scientists are striving to map colon cancer heterogeneity and to determine which factors can function as better prognostic and predictive markers for this disease. Molecular features are however sparsely assessed in current clinical practice, even though the histopathological parameters typically used do not suffice to recognise high risk patients that could benefit from alternative treatment strategies. This review will provide numerous examples of how molecular characteristics can aid in guiding clinical decision making and can improve outcome for colon cancer patients.

Over the last 10–15 years, the colon cancer research field has moved from mainly assessing mutations to measuring gene expression patterns to describe a broader spectrum of colon cancer heterogeneity. While initially used for dichotomisation into good and poor prognosis patients, unsupervised clustering methods have revealed that separation into strictly two groups does not reflect the biological diversity that exists within colon cancer. We will discuss how several layers of biological variance can be identified by distinct gene expression-based methods and will summarise how retrospective analysis of clinical trials has revealed that this variance has consequences for responses to currently used chemo- and targeted therapies. These findings can importantly be extended to preclinical models, thus this review aims to convey that these models capture colon cancer heterogeneity and can therefore be employed to optimise the efficiency of available therapies and to find novel targets for treatment.

## Standard of care for colon cancer

Current colon cancer classification, prognosis prediction and therapy decision-making is mainly based on (histo)pathological features. The tumour, lymph node, metastasis (TNM) staging system functions as the backbone and uses anatomical information to stage a carcinoma, in which predicted prognosis worsens as the stage is increased^3^. Other factors known to influence prognosis include histological differentiation grade, tumour sidedness and BRAF mutations. Poor differentiation grade is associated with poor prognosis, as is the presence of a BRAF mutation^3–5^. Right-sidedness is also linked to poorer outcome and survival, but only for a specific subgroup of right-sided cancers^6–8^. Even though these negative prognostic markers are recognised in the clinic, their presence generally does not alter the treatment given. An exception can be made in stage II where factors such as a high T stage, poor differentiation grade and a low number of examined regional lymph nodes are considered as markers for high risk of recurrence, and patients can therefore be considered for more aggressive treatment compared to other stage II patients^9^.

Generally speaking, a one-size-fits-all treatment approach is used for each TNM stage of the disease. Surgical removal of the primary tumour is the main pillar of stage I–III colon cancer treatment. High-risk stage II and all stage III patients are additionally treated with adjuvant chemotherapy if the patient’s condition allows for it; age is the primary determinant. The regimen consists of folinic acid (leucovorin) and the chemotherapeutics 5-fluorouracil (5-FU) or capecitabine, a prodrug of 5-FU, and oxaliplatin (FOLFOX)^9^. Administration of chemotherapy to stage III patients increases overall survival, but the benefit for high-risk stage II patients has been called into question^9,10^. In stage IV disease a minority of metastatic lesions qualifies for surgery with curative intentions, but the main first line treatment in the metastatic setting currently consists of chemotherapy with FOLFOX or leucovorin, 5-FU or capecitabine and irinotecan (FOLFIRI) plus bevacizumab. The EGFR-targeting antibody cetuximab is also used under the prerequisite of wildtype KRAS or BRAF status, as mutations in these oncogenes render tumours insensitive to EGFR-targeted therapy^9,11,12^. Bevacizumab and cetuximab have been simultaneously administered in the metastatic setting, combined with chemotherapy, but this approach decreases quality of life and does not improve overall survival^13^.

The major issue of the standardised staging and treatment protocol is that it fails to identify the full cohort of poor prognosis colon cancer patients that should receive adjuvant therapy, and that it fails to predict which patients benefit. Some stage II patients, for example, are not categorised as high risk patients and yet eventually present with recurrent disease. Some stage III patients relapse even though they received adjuvant chemotherapy, whereas some never relapse even when no adjuvant chemotherapy was administered^9^. Consequently, some patients are inadequately treated and some are over-treated with chemotherapeutics that come with serious side effects. Inter-tumour heterogeneity, caused by the presence of distinct mutations and differential regulation of gene expression across colon carcinomas from different patients, is a contributor to these diverse responses as we will substantiate in the next sections.

## Colon cancer inter-tumour heterogeneity

### Genetic heterogeneity in hereditary and sporadic colon cancer

Part of colon cancer heterogeneity is installed at the premalignant stage, driven by mutations and epigenetic regulation affecting distinct biological pathways. They give rise to two major classes of adenomas and carcinomas that exist in both hereditary and sporadic cases of colon cancer.

The biggest class of sporadic colon cancer contains inactivating mutations of the APC gene, detected in ~80% of the cases^14^. Somatic mutations in this gene can already be found in early lesions such as dysplastic epithelium and small, benign tubular adenomas^15^. APC was initially identified as the gene that is heterozygously mutated in the germline of patients suffering from hereditary familial adenomatous polyposis, which is associated with high lifetime incidence of colon cancer^16,17^. Taken together, these observations fuel the notion that APC loss is the initial driver event for the development of adenomas and subsequent carcinomas^14,15,18,19^. This gatekeeper role of APC can be attributed to its function in Wnt signalling, the pathway that controls the self-renewal and proliferative capacity of intestinal stem cells (ISC). Inactivation of APC through mutations or loss of heterozygosity leads to aberrant activation of Wnt signalling^18,20^, but even in the presence of dysfunctional APC, some degree of regulation of Wnt pathway activity is maintained to optimally enable adenoma formation. This is genetically determined by the location and nature of truncating APC mutations on the two alleles, and microenvironmentally by stromal production of, for example, Wnt ligands, R-spondin and hepatocyte growth factor^21–26^. Further accumulation of mutations in well-known oncogenic pathways facilitate the progression from early to high grade adenoma and ultimately carcinoma. KRAS and TP53 mutations are prevalent in early stages, and the latter can contribute to the development of chromosomal instability (CIN)^27^. The PI3K and TGFβ pathways are frequently deregulated at later stages through mutation of the PIK3CA and PTEN, and SMAD2 and SMAD4 genes, respectively^14,15,18,19^.

The second, smaller (~15%), class of colon cancer, is characterised by high mutational load caused by defective DNA mismatch repair (MMR)^14^. Repetitive DNA sequences, such as microsatellites, are especially sensitive to mutation due to dysfunctional MMR and high abundance of microsatellite length alterations, a phenotype known as microsatellite instability (MSI), is therefore used as a surrogate read-out to establish MMR deficiency^18^. A small percentage of the MSI tumours arises through genetic predisposition; individuals affected by hereditary non-polyposis colorectal cancer (HNPCC) syndrome harbour germline-inactivating mutations in MMR genes^18,28–31^. MMR deficiency in sporadic MSI tumours is usually the result of CpG island promoter methylation and hence inactivation of the MLH1 gene^14,18^, which encodes a crucial player in the MMR pathway. This promoter methylation does not stand on its own, but is accompanied by widespread methylation of promoters throughout the genome, a phenomenon known as CpG island methylator phenotype (CIMP). CIMP-high MSI tumours are furthermore characterised by the frequent presence of mutations in the BRAF oncogene^14,32–35^.

Over the last 15 years it has become more widely accepted that BRAF mutation and CIMP-high phenotype are molecular features accumulated during a distinct neoplastic pathway, the serrated pathway (reviewed in ref. ^36^). These specific changes appear to be crucial first steps in the pathway, given that they can already be detected in sessile serrated adenomas, whereas high MSI is only detected at later stages^34,37^. The early presence of CIMP and the BRAF^V600E^ oncogene might be functionally linked: it has been suggested that the BRAF oncogene directly contributes to the instalment of CIMP through direction of a repressor complex including DNA methyltransferase 3B towards CpG islands^38^. The tumour suppressor gene p16INK4a is epigenetically silenced in CIMP and this might be used to overcome BRAF-linked oncogene-induced senescence to drive progression from sessile serrated adenomas to hypermutated MSI carcinoma^39,40^.

MSI and non-hypermutated tumours can not only be distinguished using the mentioned (epi)genetic features, but also display divergent gene expression patterns, further corroborating that these carcinomas should be regarded as distinct entities^41,42^. Discrimination between the two is both prognostic and predictive for response to certain therapies. MSI is associated with a good prognos at stage I–III and these tumours do not recur or metastasise frequently. If they do, overall survival is significantly decreased compared to microsatellite stable tumours^5,43,44^. Clinical trials have revealed that MSI patients should not be treated with 5-FU as they do not benefit or as outcome is negatively affected in stage II disease^45,46^. These examples highlight that determining MSI status is clinically relevant, but unfortunately is not always standard of care. Additionally testing for the presence of the BRAF^V600E^ mutation can further distinguish between hereditary and sporadic MSI cancer, as this mutation is rarely found in the hereditary form of the disease^47^.

## Gene expression-based profiling of colon cancer

MSI status and a few mutations can be used as prognostic markers, but they fail to accurately select all poor prognosis patients. Over the last 10–15 years the focus has therefore shifted towards employing gene expression patterns as prognostic and predictive markers, an approach that captures a broader perspective on inter-tumour heterogeneity; it measures both differential activity of cell signalling pathways in cancer cells that is not necessarily regulated by mutations alone, and takes the make-up and influence of the tumour microenvironment into account.

### Colon cancer outcome prediction

Two landmark studies utilised microarrays on primary breast cancer samples to deduce a 70-gene classifier to stratify patients into good and poor prognosis subsets^48,49^. A second approach uses a quantitative reverse transcriptase PCR gene expression panel as a prognostic tool for oestrogen receptor positive breast cancer^50^. Both platforms have been adapted to predict prognosis in stage II and III colon cancer and are known as ColoPrint^®^ and Oncotype DX^®^, respectively. The latter can additionally predict benefit from treatment with 5-FU and leucovorin^51–53^. Neither of the two commercially available assays are however part of current standard clinical care for colon cancer patients^9,54^.

Over 20 additional gene signatures designed to identify poor prognosis patients have been published (summarised in ref. ^55^). Although these signatures may provide clinical use in terms of identification of patients that have higher risk of recurrence, they do not provide insight into the underlying reason. It is important to realise that recurrence for stage III patients may result from the aggressive nature of tumour cells, prompting a higher propensity for recurrence or early metastatic spread (poor prognosis), or from inferior response to treatment (poor prediction). Therefore, these signatures ignore gene regulatory information that might be relevant for understanding specific tumour biology that underlies the differential outcome.

Alternatively, more holistic molecular classification efforts have therefore been made to fully capture colon cancer heterogeneity, either using supervised approaches to study specific cell signalling pathway activity, or preferring unsupervised hierarchical clustering methods. These methods could provide insight into what biological processes fuel aggressive behaviour of cancer cells and what mechanisms are responsible for insensitivity to therapies.

### Supervised, biology-driven clustering of colon cancer

The benefit of looking at gene expression signatures rather than mutations alone to study pathway regulation is exemplified by the observations that supervised analysis can be used to detect subsets of colon cancer in which the BRAF, KRAS and/or PIK3CA genes are not mutated, but gene expression patterns nevertheless match those of mutated tumours^56,57^. Hence, adherence to this pattern can point out patients in which oncogenic pathway activation is achieved through less frequent mutations or through non-genetic mechanisms. Importantly, these tumours behave similarly as compared to their mutated peers; for instance, BRAF-like tumours are equally associated with poor prognosis, but might be sensitive to microtubule-targeting chemotherapeutics^56,58^ and EGFR-activated cancers do not respond to cetuximab, but might be sensitive to drugs targeting downstream effectors in this pathway^57^.

Other supervised efforts trying to implement biologically relevant information dichotomised colon cancer based on low or high expression of gene signatures associated with normal ISC or cancer stem cells (CSC)^59,60^. The underlying notion of this approach was that CSC fuel tumour growth and give rise to different cancer cell types in the tumour, and that high CSC signature expression functions as a surrogate measure for augmented CSC presence^20,59–64^. As in other tumour types, ISC/CSC-like expression patterns in colon cancer were associated with low overall survival rates^59,60,64^. Surprisingly, high expression of Wnt signalling target genes was associated with good prognosis, even though activation of this pathway is a hallmark of the ISC and CSC state^20,23,60^. It has therefore been suggested that adherence to the ISC/CSC signatures reflects the relatively undifferentiated nature of the tumour, rather than the abundance of Wnt-active CSC^60^. Later unsupervised clustering approaches support this notion, as they, amongst others, identify a highly Wnt active cluster and in some cases a separate stem cell-like cluster^65–69^.

### The consensus molecular subtypes of colorectal cancer

**Fig. 1:**
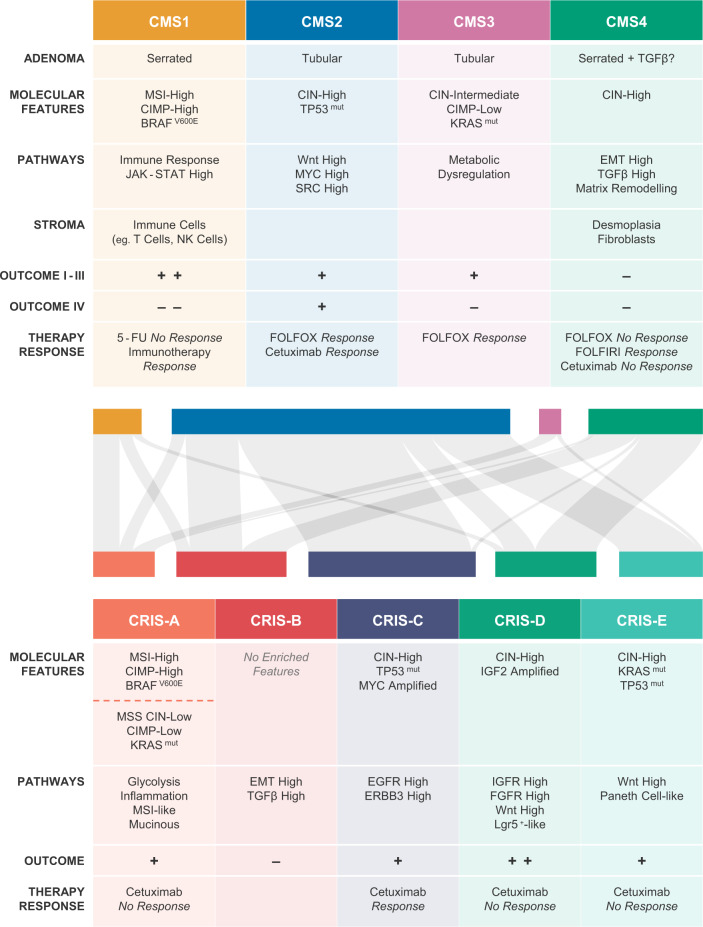
Key characteristics of CMS and CRIS subtypes and their inter relatedness. Defining features of the CMS (top) and CRIS (bottom) subtypes are summarised in the respective tables. The relationship between classification systems is illustrated by the Sankey diagram in the middle. A total of 119 established cell lines (*N* = 91) and primary cell and organoid cultures (*N* = 28) could be assigned with high confidence using both classifiers^89,90^. Colours of nodes correspond to the respective CMS and CRIS subtypes in the tables, size of the nodes reflects the number of cultures adhering to that particular subtype. For comparison with patient CMS–CRIS distribution, please refer to the publication of Isella et al.^89^.

Although supervised clustering strategies were prognostic and predictive to a certain extent, forcing all tumours into two subtypes probably does not acknowledge the full heterogeneous biology of colon cancer; MSI cancers are for example not recognised as a separate group. Early unsupervised clustering methods revealed that more than two flavours of colon cancer most likely exist, although these studies did not discuss biological mechanisms associated with each cluster, nor did they link differential gene expression patterns to prognosis and therapy response^14,35,41^. Several research groups set out to integrate this information in unsupervised clustering, leading to the separation of colon cancer into 3–6 distinct subtypes, depending on the choice of gene expression analysis platforms, bioinformatics approach and statistical analyses^14,65–71^. Although the number of subtypes identified diverged, it soon became apparent that some key biological characteristics were shared between subtypes from different studies^72^. All approaches distinguished a cluster comprised of MSI/CIMP-high tumours, a mesenchymal/stem cell-like cluster and clusters in which gene expression patterns were observed that matched patterns associated with (subsets) of epithelial cells in the normal colon. An international consortium encompassing all the researchers originally reporting on the gene expression-based classification systems, consolidated separate findings into one overarching stratification system, the CMS (summarised in Fig. 1)^73^.

Four subtypes are described that, importantly, are linked to outcome: in stage I–III colon cancer, CMS4 patients present with the poorest overall survival, and CMS1 presents with the poorest outcome in the metastatic setting, whereas survival rates after relapse are higher in CMS2 tumours^73^. These findings were further corroborated in independent clinical trials retrospectively analysing CMS status of metastatic colorectal cancer patients^74,75^.

Differential outcome can be partially explained by the biological features of each subtype: CMS1 represents the MSI/CIMP-high cancers displaying high immune cell infiltration and harbouring the majority of BRAF^V600E^ mutations present in the dataset. CMS2 and CMS3 are alike and share high expression of an epithelial signature, but differ in subtle ways. The CMS2 subtype is also termed the canonical subtype because it is characterised by high levels of CIN and high expression of Wnt and MYC target genes. KRAS mutations are enriched in CMS3. This subtype displays high activation levels of metabolic pathways and is therefore also described as the metabolic subtype. CMS4 expresses high levels of genes associated with a mesenchymal phenotype, illustrated by activation of the epithelial–mesenchymal transition (EMT) and TGFβ pathways. Mesenchymal features have been linked to poor prognosis in various other cancer types^76^. CMS4 is further characterised by high stromal content and infiltration of lymphocytic and monocytic immune cells^77^. It is important to note that while some mutations might be significantly more frequent in one subtype over the other, none are exclusively present in one CMS class. The differential gene expression signatures, linked to distinctive pathway activity between CMS subtypes, do however illustrate that the subtypes are biologically distinct and highlight that this is installed beyond the presence of genetic alterations. It furthermore makes it plausible that they can be targeted with specific classes of drugs as discussed later.

It has been suggested that biological differences between CMS subtypes are in part installed at the premalignant state. Expression patterns of SSA obtained from serrated polyposis syndrome patients have been connected to the CMS1 and CMS4 subtypes, whereas tubular adenomas from familial adenomatous polyposis patients bear resemblance to the epithelial subtypes^66,78^. Other classification efforts on precursor lesions have been described and categorise most hereditary and sporadic tubular adenomas into the epithelial subclasses CMS2 and CMS3, depending on the strategy employed. The majority of sporadic and serrated polyposis syndrome-derived sessile serrated adenomas and hyperplastic polyps classify as CMS1 in these analyses and CMS4-like adenomas are detected at very low incidence^79,80^. This discordance suggests that although mesenchymal features may be apparent in sessile serrated adenomas, they are not as pronounced as in CMS4 cancers. We therefore suggest that while tubular and sessile serrated adenomas may be predisposed to transform into a carcinoma of a particular CMS subtype, further oncogenic transformation and microenvironmental cues during progression are needed to definitively install the biological programs associated with the individual subtypes. One of those cues might be high levels of TGFβ that push sessile serrated adenomas towards a more CMS4-like rather than a CMS1-like state^78^.

Interestingly, the patterns in activity of specific biological pathways associated with each CMS subtype can be extrapolated to subtypes identified in carcinomas of other gastrointestinal origin. Elaborate discussion of this observation goes beyond the scope of this review, and it has been diligently reviewed elsewhere^76^. We would however like to point out an important message that can be distilled from this: these tumours originate from unique tissue types, each using organ-specific gene expression programs and each influenced by microenvironments composed of varying cell types. The overlap in subtype characteristics could point to epithelial cells using specific routes towards the development into carcinoma cells that lead to similar behaviour of these tumours. Additionally, it creates an opportunity to use the same treatment regimen for tumours originating from different tissues that nevertheless adhere to a similar subtype. An alternative explanation for the overlap in subtype characteristics across tumour types is that it does not represent distinct biological behaviour of the epithelial cancer cell compartment, but rather reflects the attraction of a unique microenvironment consisting of different cell types.

### Caveats of the CMS and alternative classification strategies

It is evident that subdivision of colon cancer in CMS is not only driven by gene expression profiles derived from the tumour cells, but that it is highly influenced by the abundance and composition of the microenvironment. This is especially apparent in CMS1, in which relatively high numbers of immune cells infiltrate the tumour, and in CMS4 where the high expression of EMT and TGFβ target genes can in part be attributed to an ongoing desmoplastic reaction recruiting high amounts of fibroblasts^73,81–83^. The dependence of the classification on the microenvironment also implies that the sampling site of the tumour piece may impact on CMS classification, as the stromal make-up can differ between specimens. It has indeed been noted that CMS assignment is discordant between samples obtained from the same patient, which holds true when comparing multiple biopsies of the primary tumour, samples acquired from the invasive front and tumour core, or from primary and metastatic sites^84–87^.

Partial reliance on stromal gene expression for CMS classification is furthermore illustrated by the fact that stratification of preclinical models is complicated by the absence of stromal cells. The gene expression profile of cancer cell lines is solely derived from epithelial cells, while the stromal compartment of patient-derived xenografts (PDX) is derived from immune-deficient mice that lack B-, T- and often NK-cells. Depending on the platform used, the murine contribution can be partially detected (array-based analysis is optimised for human sequences) or can be analysed separately (RNAseq)^88–90^. Nevertheless, assignment of all subtypes, including the mesenchymal ones, to large cancer cell line panels has been achieved by multiple groups. Irrespective of the distinct bioinformatics analyses used, the majority of cell lines or primary cultures are concordantly subtyped, and switches in classification usually occur between the related CMS2/CMS3 and CMS1/CMS4 subtypes^66,90–92^. Taken together, this indicates that CMS-associated biology is still captured in these models. Similarly, assigning CMS to PDX models is feasible although this proved to require further adaptation of the bioinformatics method. It has been reported that if the patient-derived CMS random forest classifier is applied, PDX models are not classified as CMS4, specifically because murine stromal signals are not measured effectively^81,82,89,93^. Nevertheless, this can be overcome by rebuilding classifiers enriched for genes expressed in the epithelial fraction, and these adapted classifiers perform well on tumours from patients and on PDX models. In addition, such epithelial-enriched classifiers actually stratify cell lines more optimally compared to the CMS random forest classifier alone^90,92,94^. This implies that specific CMS features are locked in the tumour cells and that these cells are likely responsible for the manipulation of the microenvironment, yielding stromal-rich tumours in CMS4 or immune cell-rich tumours in CMS1. In agreement, the validity of these PDX classifications was further substantiated by the observation that tumours with a given subtype as defined for the patient’s specimen maintained their subtype in the PDX model in the majority of the cases.

Such a shift in focus towards cancer cell intrinsic expression patterns in the tumour setting has been proposed to resolve some of the issues associated with CMS classification. Gene expression data acquired from whole tumour samples however does not allow for distinction between cancer cell and stroma-derived signals. Single cell RNAseq can resolve this issue in the future, but researchers reasoned that the unique composition of PDX, with human cancer cells and mouse stroma, would in the meantime allow for identification of signals solely expressed in the tumour epithelium by employing two bioinformatical filtering strategies. Firstly, microarray probes cross-hybridising with murine transcripts were not included in further analyses as signals from such probes cannot be assigned faithfully to human or mouse RNA. To further enrich for transcripts of epithelial origin, RNA sequencing data was obtained for a number of PDX. If more than 50% of the total expression of a gene originated from mouse transcripts, it was excluded in the downstream analysis^89^. These two filtering strategies allowed for unsupervised stratification of colon cancer into five CRC intrinsic signature (CRIS) subtypes that capture epithelial-specific gene expression profiles driven by oncogenic mutations and pathways (summarised in Fig. 1).

CRIS-A consists of KRAS-mutated samples and MSI, BRAF-mutated samples and is characterised by glycolytic metabolism and inflammatory signals. CRIS-B tumours are poorly differentiated and display active TGFβ and EMT signalling. CRIS-C encompasses CIN tumours with wildtype KRAS status, MYC amplification and increased EGFR pathway activity. CRIS-D is associated with high Wnt, fibroblast growth factor receptor (FGFR) and insulin-like growth factor receptor (IGFR) pathway activity, of which the latter is in part achieved by IGF-2 amplification. CRIS-E is likewise characterised by high Wnt activity and TP53 mutations are prevalent in this subtype. Significant overlap exists between CRIS and CMS subtypes, although differences are described (Fig. 1). CRIS-A is mainly constituted of CMS1 and CMS3 tumours. CRIS-B incorporates the other CMS1 samples and a subset of CMS4 samples. The rest of the CMS4 tumours are equally distributed over CRIS-C, CRIS-D and CRIS-E, as are the CMS2 tumours^89^. Importantly, the CRIS classifier is better suited to concordantly classify multiple samples obtained from the same patient, irrespective of the sampling site, as its focus on the tumour cell intrinsic phenotype reduces the influence of stromal constitution^85,86^. Consequently, different preclinical models, such as cell lines or PDX models, can also be robustly classified without adapting the CRIS gene set^89^. As illustrated in Fig. 1, distribution of colon cancer cell lines over the respective CRIS and CMS classes follows the trends observed in patient samples, although small differences exist.

The CRIS classification system is thus fitted to capture oncogenic driver programs specifically active in the cancer cell compartment of a tumour. As will be discussed in the next section, this provides insight in therapy sensitivities, but filtering out stromal contribution to gene expression patterns in tumours comes with drawbacks as well. This is firstly illustrated by the observation that the prognostic value of CRIS is improved when information about the abundance of fibroblast infiltration is added, which to some extent results in a strategy reminiscent of the identification of the poor prognosis, stromal-rich CMS4 subgroup^89^. Furthermore, although the derivation of the CRIS classifier based on PDX allows for distinguishing cancer cell intrinsic features, it also introduces a bias due to two inherent limitations: (i) signalling incompatibility exists between murine and human ligands/receptors, and certain biological programs and feedback loops between the compartments are thus not completely recapitulated in PDX, and (ii) not all tumours successfully establish a PDX and it has been reported that some CMS classes are more capable of forming serially transplantable xenografts compared to others^95^. It must also be considered that predominant expression of a gene in the stroma does not mean it cannot be differentially expressed in the cancer cell compartment, and overall exclusion of such genes from further analysis can also neglect relevant biology.

### Colon cancer heterogeneity is captured in preclinical model systems and is linked to differential therapy responses

Established cell lines and genetically engineered mouse models (GEMM) traditionally comprised the model systems available for preclinical research, but recent advances allow for generation of different patient-derived models. We have already alluded to direct PDX, in which a piece of human tumour is transplanted in immunocompromised mice^96^. Patient material can also be used to establish primary cell lines grown in suspension^62^, and seminal work has described protocols for in vitro 3D culture and expansion of both mouse and human organoids derived from healthy and tumour tissue^97,98^. Both culture types can be grafted into immune-deficient mice to procure indirect PDX. Furthermore, step-wise introduction of mutations in driver genes in organoids from healthy tissue can be used to model the full spectrum of adenoma-to-carcinoma sequence, including metastasis^27,99–104^.

**Fig. 2:**
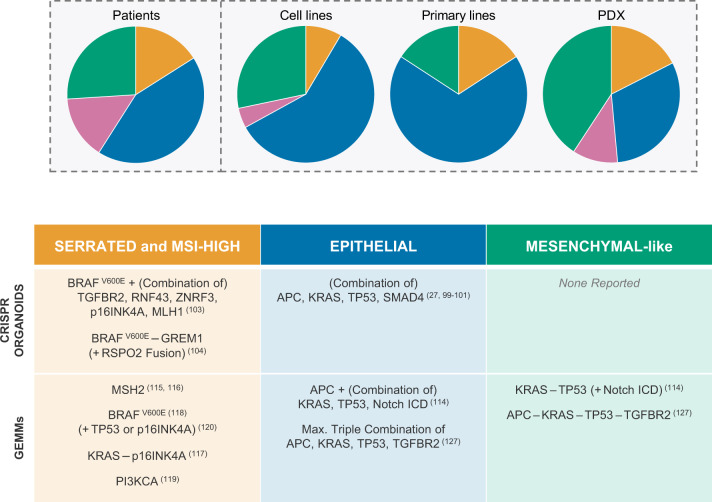
CMS subtypes in preclinical models. Top: Pie charts illustrating distribution of CMS subtypes in different preclinical models compared to distribution amongst patients as reported in Guinney et al.^73^. Classifier used for cell lines is the support vector machine classifier developed and trained as described in Linnekamp, van Hooff et al.^91^. Datasets used for cell lines: GSE36133, GSE100478, GSE59857 and GSE68950. Datasets for primary cell lines: GSE100549 and GSE100479 supplemented with additional primary spheroid culture gene expression profiles generated by RNAseq in the laboratory of Prof. Dr. Giorgio Stassi in Palermo (unpublished data). (Primary) cell lines were allocated to a certain CMS class using the following rules: (i) consistent CMS class prediction across all datasets with probability score >0.4. (ii) Consistent CMS class prediction across all datasets with probability score >0.5 in 33% of datasets and >0.35 in all other datasets. (iii) Probability score >0.5 for one consistent CMS class in 66% of the datasets. CMS class prediction in other datasets could differ from majority, but with probability score <0.5. (iv) Probability score cut-off was set to >0.5 if cell line was present in a single dataset. PDX classification and distribution obtained from and implemented according to Prasetyanti et al.^95^. Bottom: Overview of reported CRISPR-edited organoids and genetically engineered mouse models that reflect distinct biology of human adenomas and colon carcinomas. Numbers in between parentheses refer to original publication.

Importantly, all these different types of preclinical models capture the various facets of colon cancer heterogeneity, including CRIS and CMS subtypes, illustrated by a vast range of published repositories^65,66,90–93,95,100,101,105–112^. Before we discuss patterns in therapy sensitivity and resistance that have been identified, we would like to point out that every preclinical model is associated with a certain level of bias in terms of the subgroups that it represents best (Fig. 2). GEMM for colon cancer were typically driven by APC loss and these mice develop tubular adenomas and epithelial-like tumours^113,114^. Novel conditional GEMM have emerged over the last years in which MMR genes are deleted to model MSI colon cancer^115,116^ or in which APC is not included as a driver gene. These non-canonical GEMM typically give rise to serrated-like adenomas and more invasive and aggressive colon cancer^114,117–120^. Traditional cell lines are enriched for MSI positivity, and primary cell line and organoid cultures adhere more to the CMS2 subtype^90^. Other patient-derived models are likewise associated with biases: the original organoid establishment protocol is less suitable for generating serrated polyp cultures^98,112^, whereas direct PDX appear to be more successfully derived from CMS1 and CMS4 tumours^95^. Fully individualised therapy selection based on an in vitro or in vivo model is therefore still challenging, as one cannot know up front what technique would establish a personal preclinical model for a particular patient. Studying the responses across panels of models has however yielded valuable insights.

Initial clinical trials using vemurafenib to target the BRAF^V600E^ oncogene failed in colon cancer, despite its effectiveness in melanoma^121^. Preclinical data revealed that feedback activation of the MAPK pathway through EGFR was the underlying mechanism of resistance, and recent trials reported that combination of vemurafenib with MAPK pathway inhibitors overcomes resistance^122–124^. Various mechanisms leading to cetuximab insensitivity were elicited in PDX, amongst which mutations in various components of MAPK signalling (upstream receptor tyrosine kinases, PTEN, PI3K, BRAF, KRAS and NRAS)^91,93,111^. Pathway hyperactivation through amplification or upregulation of ligands and receptors is also frequently observed, such as human epidermal growth factor receptor 2, hepatocyte growth factor receptor and FGFR1 amplification and IGF2 overexpression^110,111,125,126^. Drugs to target these compensation mechanisms are available, and can indeed be successfully combined with cetuximab to generate better responses^111,126^. As with drug resistance, patterns can be observed in cell lines and PDX that predict sensitivity to cetuximab, including high Wnt and Myc pathway activity.

It should be noted that the described escape mechanisms and indicators for sensitivity signify specific CMS and CRIS subtypes, in patient samples and in preclinical models. CMS2-like and CRIS-C tumours display high Wnt and Myc activity and are associated with cetuximab response. A subgroup of CMS2 classifies as CRIS-D in which the FGFR and IGF signalling pathways are activated, and these samples do not respond to cetuximab^66,73,89–92^. CMS4-like cell lines do not respond to cetuximab either and are additionally associated with poorer responses to chemotherapy with 5-FU and oxaliplatin^66,90,92^.

The discussed examples focus on tumour cell intrinsic differences in pathway activation that are recapitulated in model systems. Increased stromal infiltration in the CMS4 subtype is similarly translated to PDX models^90^, suggesting that CMS4 cancer cells actively attract this microenvironment and that the tumour–stroma interaction could pose as a targeting possibility. Two recent studies using novel mouse models that give rise to primary tumours and spontaneous metastases support this notion^114,127^. The aggressive tumours were characterised by presence of reactive stroma, stimulated by TGFβ, and activation of CMS4 and CRIS-B-associated gene expression pathways. Critically, inhibition of TGFβ decreased metastatic outgrowth and caused a switch from an immunosuppressive microenvironment to one that allows for T-cell infiltration. The existence of an immunosuppressive environment has been noted before in CMS4 cancer specimens^77,87^ and these mouse studies suggest that TGFβ inhibitors can be combined with immune checkpoint inhibition to elicit an immune response^114,127^. They furthermore reveal that studying the tumour–stroma interaction is an avenue worth exploring to infer additional candidates for targeted therapy^128^. In vitro co-culture experiments, dissection of mouse and human RNA expression patterns in PDX models through RNA sequencing or single cell RNA sequencing of human tumours can be used to reveal these interactions.

## Translation of CMS to the clinic

A number of criteria still need to be met to warrant implementation of CMS subtyping in clinical practice. First, the laboratory and informatics procedures currently employed for classification need to be improved and adapted to better suit the clinical reality. Second, the predictive value of CMS for therapy response needs to be corroborated by (prospective) clinical trials. In this section, we aim to shed light on the technical challenges faced to robustly classify patient samples and the avenues that are currently explored to resolve these issues. We will then give an overview of what we have learned so far about therapy outcome prediction by retrospective analysis of clinical trials.

### Optimisation of CMS classification for clinical applicability

Gene expression profiling is readily influenced by the practical methods used to obtain and prepare RNA for analysis and is affected by the platform chosen to generate the expression profile. Normalisation issues additionally complicate CMS single sample prediction^73,129,130^. Furthermore, the majority of the RNA samples obtained for the CMS discovery datasets was extracted from fresh frozen tumour samples, whereas clinical specimens are normally formalin-fixed and paraffin-embedded (FFPE) before storage. The FFPE process generates low quality RNA due to fragmentation and degradation over time and these shorter fragments are more difficult to quantify using RNAseq, microarray or PCR-based techniques, but novel technology offers a solution to enable expression profiling on FFPE material. The NanoString platform uses short probes (35–50 nucleotides) to capture and quantify mRNA sequences and is therefore more compatible with fragmented RNA. Three recent studies have built customised NanoString probe sets and successfully classified panels of FFPE material according to CMS^75,87,131^. CMS classification between patient-matched FFPE and fresh frozen material was concordant, although sample size was limited^75,87^. Formal cross-validation of newly developed NanoString classifiers and the original consortium random forest classifier will have to be extended to a larger panel of matched samples to fully support the transformation towards a NanoString-based CMS classification protocol.

To aid the transition to the use of FFPE material, others have also assessed if immunohistochemistry for CMS-specific markers (CDX2, FRMD6, HTR2B and ZEB1) allows for classification. Combining these markers with pan-cytokeratin staining and MSI status facilitates stratification into CMS1, epithelial CMS2/CMS3 and mesenchymal CMS4 classes. The added benefit of immunohistochemistry is that the pathologist can specifically look at protein expression in the cancer cell fraction, thereby reducing the influence of stromal infiltration on CMS classification. Non-concordant CMS assignment of multiple biopsies from the same patient might thereby be overcome, but it then needs to be established if staining intensity for the CMS markers is similar in multiple regions of the same tumour^86^. Along that line of thinking, immunohistochemistry on FFPE material and review by a pathologist should be incorporated in the CMS classification allowing for a more consistent RNA isolation protocol. Preferably, CMS testing should be performed on sections that are routinely used for pathological diagnosis.

### Therapy efficacy in subtypes: discovery in preclinical setting and retrospective analysis of trial samples

Over the last few years CMS(-affiliated) subtyping has been retrospectively applied to publicly available patient datasets and clinical trial cohorts to define predictive markers for therapy efficacy (summarised in Table 1)^66,67,71,74,75,132–137^. We are aware of the fact that the majority of the used cohorts consist of metastatic colon cancer patients and differences between datasets (patient characteristics, trial inclusion and exclusion criteria, treatment composition) and methods used for CMS classification exist. For an extensive discussion of the various intricacies that should be considered for the interpretation of these studies, we would like to point to refs. ^138–140^. Nevertheless, clinically relevant patterns in terms of therapy response can be distilled from these analyses, and we will highlight them in this section.

**Table 1:** Summary of literature addressing the association of CMS(-like) subtypes with therapy response.

Therapy composition	Reference	Trial/Cohort	Samples	Stage	Treatment	Main results
Chemotherapy only	Roepman et al.^71^	Local cohort	222	Stage III	No adjuvant therapy vs. 5-FU-based therapy	Epithelial B-type benefits from chemotherapy, mesenchymal C-type does not.
	Song et al.^141^	NASBP C-07	1729	Stage II and III	Leucovorin and 5-FU ± Oxaliplatin	Addition of oxaliplatin only benefits CMS2(-like) patients
	Okita et al.^145^	Local cohort	193	Metastatic	Irinotecan-based vs. Oxaliplatin-based	CMS4 benefits from irinotecan-based therapy
	Allen^a^, Dunne^a^ et al.^142^	Local cohort Marisa et al.^68^	156, 479	Stage II and III	No adjuvant therapy vs. 5-FU-based therapy	Only stage II and III CMS2 and stage III CMS3 benefit from adjuvant chemotherapy
Cetuximab	De Sousa e Melo^a^, Wang^a^ et al.^66^, Sadanandam et al.^67^	Khambata-Ford^a^, Garrett^a^ et al.^132^	110	Metastatic	No therapy vs. cetuximab mono therapy	No benefit for cetuximab in KRAS wildtype mesenchymal CCS3/stem-like subtypes, only in CCS1/TA-like epithelial subtypes.
Chemo- plus targeted therapy	Mooi et al.^74^	AGITG MAX	237	Metastatic	Capecitabine vs. Capecitabine + Bevacizumab (± Mitomycin)	Bevacizumab use only benefits CMS2 and possibly CMS3.
	Smeets et al.^134^	ANGIOPREDICT, CAIRO^149^	204, 205	Metastatic	Fluoropyrimidine-based chemotherapy ± Bevacizumab	CIN-intermediate/high (enriched for CMS2 & CMS4) benefit from bevacizumab, CIN-low (enriched for CMS1 & CMS3) do not.
	Trinh et al.^137^	CAIRO2^13^	311	Metastatic	Capecitabine + Oxaliplatin + Bevacizumab ± Cetuximab	Benefit cetuximab only observed in KRAS wildtype epithelial (CMS2 & CMS3) group, not in mesenchymal (CMS4) group.
	Stintzing et al.^135,136^	FIRE3	315	Metastatic	FOLFIRI + Bevacizumab or + Cetuximab	Cetuximab yields more benefit than bevacizumab in CMS4.
	Lenz et al.^75^	CALGB/SWOG 80405	581	Metastatic	~75% FOLFOX, ~25% FOLFIRI + Bevacizumab or + Cetuximab	Bevacizumab yields more benefit than cetuximab in CMS1. Cetuximab yields more benefit than bevacizumab in CMS2.

Main characteristics of studies are listed, as well as the most notable outcomes. Superscripted numbers refer to original publication.

vs. versus.

^a^Indicates shared first authorship.

Not all stage II and III colon cancer patients respond to adjuvant chemotherapy, with recent data suggesting that CMS subtype-specific sensitivities can potentially explain this variation. Multiple studies have pointed out that 5-FU-based therapies, also when supplemented with oxaliplatin, only benefit epithelial CMS2-like patients and not those adhering to a CMS4-like subtype^71,141,142^. Given the poor prognosis that is already associated with the CMS4 subtype, these clinical studies call for consideration of other chemotherapeutics to increase overall survival. A potential successful alternative was suggested for the stem-like colon cancer subtype, which is reminiscent of the CMS4 subtype. At metastatic disease these tumours appeared more sensitive to FOLFIRI, in which oxaliplatin is replaced for irinotecan^67^. Although available sample sizes of patients were initially too small to fully support the potential of irinotecan use^143,144^, two independent clinical trials with larger patient cohorts have since substantiated the enhanced effect of FOLFIRI over FOLFOX in CMS4 in the metastatic setting^133,145^.

Metastatic patients are currently treated with FOLFIRI or FOLFOX supplemented with targeted therapies bevacizumab or cetuximab, depending on KRAS mutation status^9^. Clinical trials and preclinical studies have however described that further separation of patients or models according to CMS subtype is relevant for predicting cetuximab efficacy. Patients adhering to a mesenchymal subtype do not benefit from monotherapy with cetuximab regardless of the KRAS mutation status^66,91–93,132^. Combining cetuximab with FOLFOX does not provide better results; overall survival is not increased in CMS4-like patients^75,137^. Another trial however revealed opposing results, as CMS4 patients responded better to cetuximab addition than to bevacizumab, under the condition that FOLFIRI forms the chemotherapeutic backbone^136^. It remains to be elucidated whether the conflicting outcomes between trials are, in part, caused by the higher sensitivity of CMS4 to FOLFIRI compared to FOLFOX^133,139,145^. It however underlines that the best combination of chemotherapy plus bevacizumab or cetuximab can be different for each CMS subgroup. Preclinical models can help in defining the best combination for each subtype in order to guide the design of future clinical trials.

The benefit of bevacizumab over cetuximab in MSI/CMS1 classes of tumour is also not supported by all trials^74,75,134^, although the hypermutated status and abundant immune infiltration observed in these tumours has made them candidates for immune checkpoint inhibition. Treatment of metastatic MSI colorectal cancer patients has yielded good therapy responses in a subset of patients, and recent genetic analysis of tumours has revealed that a higher load of genomic insertions and deletions due to MMR deficiency can be used as a selective marker for better therapy response^77,146,147^.

Taken together these results demonstrate that CMS classification can be used to explain differences in response to the therapies currently used in the clinic and, critically, that poor prognosis CMS4 tumours tend not to respond.

## Concluding remarks

To summarise, colon cancer is a heterogeneous disease marked by various molecular features such as mutations, CIN, MSI status and gene expression patterns. These factors are mostly disregarded in the clinic and we have pointed out that they should be incorporated in clinical decision making, as they are relevant for prognosis and therapeutic response.

The CMS and CRIS classification strategies integrate differential activity of biological programs between tumours beyond single gene mutations, and can importantly be linked to therapy sensitivity or resistance in specific subtypes, making them an attractive method to stratify colon cancer patients.

Continued efforts to devise a standardised CMS classification method that functions reliably on FFPE material are being made. Its completion would aid translatability to the clinic, although formal integration would only be warranted if clinical trials support the added value of CMS classification for patient prognosis or prediction of therapy response. To fulfil that need, CMS classification should be applied retrospectively to trials, but it should additionally be incorporated in prospective studies. In that regard the colon cancer field can learn from the i-SPY 2 trial that segregates breast cancer patients in 10 distinct molecular subgroups, adapts the treatment plan accordingly and that allows for rapid influx of experimental drugs into the trial^148^. Prospective studies taking CMS stratification into account are currently being set up. We have discussed that preclinical models capture the heterogeneous biology of colon carcinomas and we therefore believe that they should be employed to design novel treatment strategies that could yield better results than the current standards. The most promising findings should eventually be tested in these prospective trials. This will ultimately facilitate moving beyond the one-size-fits-all treatment currently used and may hopefully improve disease outcome for more colon cancer patients.

## Abbreviations

5-FU: 5-Fluorouracil
APC: Adenomatous polyposis coli
CIMP: CpG island methylator phenotype
CIN: Chomosomal instability
CMS: Consensus molecular subtypes
CRIS: CRC intrinsic subtypes
CSC: Cancer stem cell(s)
EGFR: Epidermal growth factor receptor
EMT: Epithelial-mesenchymal transition
FFPE: Formalin-fixed paraffin-embedded
FGFR: Fibroblast growth factor receptor
FOLFIRI: Folinic acid (Leucovorin), 5-FU or capecitabine (5-FU prodrug) and irinotecan
FOLFOX: Folinic acid (Leucovorin), 5-FU or capecitabine (5-FU prodrug) and oxaliplatin
GEMM: Genetically engineered mouse model(s)
IGF(R): Insulin-like growth factor (receptor)
ISC: Intestinal stem cell(s)
MAPK: Mitogen-activated protein kinase
MMR: DNA mismatch repair
MSI: Microsatellite instability
PDX: Patient-derived xenograft(s)
PI3K: Phosphoinositide 3-kinase
TGFβ: Transforming growth factor beta
TNM: Tumour, lymph node, metastasis
